# Routine Multiple Duodenal Biopsy during Endoscopy of Dyspeptic Patients Seems Unnecessary for Screening of Celiac Disease

**DOI:** 10.1155/2020/6664741

**Published:** 2020-12-22

**Authors:** Amir Behforouz, Abbas Esmaeelzadeh, Homan Mosanan Mozaffari, Ali Mokhtarifar, Elham Faravani, Sakineh Amoueian, Alireza Khooei, Lida Jarahi, Ladan Goshayeshi

**Affiliations:** ^1^Radiology Department, Faculty of Medicine, Mashhad University of Medical Sciences, Mashhad, Iran; ^2^Gastroenterology Department, Imam Reza Hospital, Mashhad University of Medical Sciences, Mashhad, Iran; ^3^Pathology Department, Mashhad University of Medical Sciences, Mashhad, Iran; ^4^Department of Community Medicine, Mashhad University of Medical Sciences, Mashhad, Iran

## Abstract

**Introduction:**

Celiac disease (CD) is a chronic and common cause of dyspepsia with a rising prevalence worldwide. This study is aimed at investigating the prevalence of CD in dyspeptic patients based on serology and biopsy, determining the associated factors, and assessing the necessity of regular duodenal biopsies from normal mucosa in diagnosis of CD among dyspeptic patients.

**Methods:**

This cross-sectional study was performed on 530 adult dyspeptic patients who underwent gastroduodenoscopy in Imam Reza hospital, Mashhad, during 2016-2018. Demographic characteristics, clinical data, and laboratory analyses were extracted from hospital records. CD was diagnosed based on intestinal biopsy and serum antitissue transglutaminase (anti-TTG) levels. Mucosal lesions were classified according to the modified Marsh classification. Data were analyzed in SPSS with *P* < 0.05 being considered significant.

**Results:**

Overall, 163 males (30.8%) and 367 females (69.2%) with an average age of 46.38 ± 15.54 years were studied. High anti-TTG levels were seen in 36 (6.8%) patients, and duodenal pathologies were seen in 23 (4.5%) patients. Fifteen (2.8%) were diagnosed with CD based on both serology and biopsy. Bloating was the most common type of dyspepsia in CD patients (7, 46.7%), followed by epigastric pain (6, 40%), and postprandial fullness (2, 13.3%). Two CD patients (13.3%) reported a positive family history for CD. Logistic regression model showed that iron deficiency anemia (IDA), anti-TTG level, and Helicobacter pylori infection were predictors of histological changes of CD, whereas IDA was the only independent predictor of CD in dyspeptic patients (OR = 17.65, 95%CI = 1.53‐202.52, and *P* = 0.021).

**Conclusion:**

CD is prevalent in dyspeptic patients, but routine biopsy from normal-appearing duodenal mucosa is not recommended for all patients. Serological studies, complete history, and careful endoscopic evaluation may provide better cost-effective clinical solutions to improve the diagnostic yield of celiac disease in dyspeptic patients.

## 1. Introduction

Celiac disease (CD) is known to be caused by an immune reaction to dietary gluten (a storage protein in wheat, barely, and rye), primarily affecting the small intestine. CD is a common cause of chronic malabsorption of fat-soluble vitamins, iron, and potentially B12 and folic acid. CD can present itself with a variety of gastrointestinal and hepatic symptoms, while many patients are asymptomatic [1, 2]. CD also commonly causes atypical symptoms such as bloating, nausea, and vomiting [3, 4], which frequently delay diagnosis [1, 2]. It is worth nothing that overlooked CD can lead to a number of long-term complications such as metabolic bone diseases, malignancies, and even mortality [5]. CD is reported to affect about 1% of the general population in western countries [6, 7]. The advent of highly sensitive serological tests in recent decades, along with a higher suspicion of the disease, has led to a marked increase in CD diagnosis [8, 9]. The diagnosis mainly consists of serological screening of antitissue transglutaminase (anti-TTG) level and upper gastrointestinal endoscopy with multiple duodenal biopsies [10].

Dyspepsia is one of the most common gastrointestinal disorders in the general population with a reported prevalence of about 40% [2, 11, 12]. CD can present with dyspepsia in up to 24.6% of patients with CD [13]. The prevalence of CD in dyspeptic patients is reported to be 0.5-2%, which is two to nine times higher than that of the general population [14–18]. On the one hand, there is insufficient evidence to support the decision to perform multiple duodenal biopsies in dyspeptic patients. On the other hand, most dyspeptic patients do not undergo serological screening for CD [2]. Moreover, the approaches of gastroenterologists vary widely in performing duodenal biopsy for screening of CD in dyspeptic patients [19]. Latest American College of Gastroenterology (ACG) guidelines state that obtaining routine biopsies of normal-appearing duodenal mucosa for diagnosis of CD is not required unless flatulence, anemia, or diabetes is present. However, this recommendation needs more evidence [1].

Few studies have investigated the prevalence of CD in Iranian patients with persistent dyspepsia based on both serological and histological findings. This study is aimed at investigating the prevalence of CD in dyspeptic patients based on both serology and histology, finding out the factors associated with CD in these patients, and assessing the necessity and efficacy of routine biopsies from normal-appearing duodenal mucosa in detecting CD in dyspeptic patients.

## 2. Materials and Methods

### 2.1. Settings and Approval

This cross-sectional study was conducted on adults complaining of dyspepsia who underwent gastroduodenoscopy in Imam Reza hospital, Mashhad, Iran, during 2016-2018. The study protocol was approved by the Ethics Committee of Mashhad University of Medical Sciences (approval code: IR.MUMS.FM.REC.1395.235). Informed consent was obtained from all patients before they enter the study.

### 2.2. Patients

Keeping an alpha of 0.05 and a *d* = 0.024, considering the prevalence of celiac disease among dyspeptic patients in Iranian population, the required sample size was calculated to be 530 patients [15]. Patients who referred to our gastroenterology clinics with chief complaint of dyspepsia during 2016-18, underwent gastroduodenoscopy, and provided informed written consent were recruited using nonrandom available sampling method. All patients were registered in our registry database at the Gastroenterology ward of Imam Reza hospital, and their records were kept for and further follow-ups. Patients who did not incline to participate were excluded from the study.

### 2.3. Data Collection

Demographic data (including sex and age), clinical presentation (symptoms and type of dyspepsia), complete medical history of comorbidities, and familial history of celiac disease were gathered in the checklists for all patients.

All patients underwent gastroduodenoscopy in a single center by expert gastroenterologists under the same standard conditions, and six biopsy samples were taken from the duodenal mucosa in all of them based on 2013 ACG guidelines [1]. Endoscopic features of CD were categorized in four groups of atrophic duodenal mucosa with loss of folds, scalloping, nodular changes, and fissuring [20]. Results of upper gastrointestinal endoscopy also including *Helicobacter pylori* infection were compiled in the checklists.

A single expert gastrointestinal pathologist, who was blind to the results of gastroduodenoscopy, performed the histopathological study of all samples. Histological diagnosis of CD was made based on the presence of intraepithelial lymphocytes, hyperplasia of crypts, and/or atrophy of the villi. Results of the histopathological study were classified as absence of CD (degree 0) or suggestive of CD (degrees I to III) according to the modified Marsh criteria [16]. The findings of histopathological study were also recorded in the checklists.

Blood samples (5 ml) were collected from all patients. Complete blood count (CBC), serum iron, ferritin, total iron binding capacity (TIBC), 25-OH-vitamin D3, and anti-TTG immunoglobulin A (IgA) were measured according to standardized methods (using a Diametra kit in a single laboratory) [21]. Iron deficiency anemia (IDA) was defined based on the World Health Organization criteria [22]. Vitamin D deficiency was defined as the respective levels below 20 ng/ml, and vitamin D insufficiency was defined as a serum vitamin D level of 21–29 ng/m based on the Endocrine Society guidelines [23].

### 2.4. Statistical Analysis

Data were analyzed using SPSS for windows, version 20, IBM Statistics (Chicago, IL, USA). Chi-square test, Fisher's exact test, independent samples *t*-test, Mann-Whitney *U* test, one-way ANOVA test, or Kruskal-Wallis test was used to compare variables in different groups. Binary logistic regression analysis was used to assess the associations between CD and independent variables. Odds ratio (OR) and 95% confidence interval (CI) were used to present the findings of regression analyses. A *P* < 0.05 was considered statistically significant.

## 3. Results and Discussion

### 3.1. Results

Overall, 530 dyspeptic patients with a mean age of 46.38 ± 15.54 years were included, of whom 163 (30.8%) were male and 367 (69.2%) were female. High anti-TTG levels, suggestive of CD, were seen in 36 (6.8%) patients. Pathological features of the obtained duodenal samples were compliant with grades I-III of modified Marsh criteria, suggestive of CD, in 23 (4.5%) patients. Only 15 patients (2.8%) had both the serological and histopathological criteria and were definitively diagnosed as CD.

Table 1 elaborates the findings regarding the comparison of CD and non-CD patients. As the table implies, the groups had no significant difference regarding the demographic characteristics (*P* > 0.05). However, CD patients were found to have higher frequency of positive family history for CD in their first-degree relatives, compared with non-CD patients (13.3% vs. 0.2%, *P* < 0.01). Bloating was the most common manifestation of dyspepsia among the CD patients (7, 46.7%), while the respective figure in non-CD patients was epigastric pain (*P* = 0.09).

Upper gastrointestinal endoscopy revealed abnormal appearance of duodenal mucosa in all CD patients (15, 100%), while only 121 (26%) of non-CD participants had abnormal findings in gastroduodenoscopy (*P* < 0.01). According to modified Marsh classification for histopathological changes in the mucosa, among the 15 CD patients, 3 (20%) were in grade I, another 3 (20%) were in grade II, and 9 (60%) were in grade III. CD patients showed a significantly higher rate of mucosal atrophy in the sample taken from duodenum (*P* < 0.01). There was no significant difference between CD and non-CD patients regarding the presence of *H*. *pylori* infection in the biopsy specimens (Table 1).

Mean serum anti-TTG levels were significantly higher in CD patients than the respective figure in non-CD participants (*P* < 0.01). Abnormal serum levels of anti-TTG were found in 21 non-CD patients (4.1%), while all 15 CD patients (100%) had abnormally high anti-TTG levels (*P* < 0.01). There was no significant association between the level of vitamin D and CD (*P* = 0.69). Moreover, the frequency of vitamin D deficiency was not significantly different between CD and non-CD patients (*P* = 0.32).

Mean serum levels of ferritin and hemoglobin in patients with CD were significantly lower compared with the non-CD group, while the total iron binding capacity was significantly higher in the CD patients (*P* < 0.01). Of the 40 cases with IDA, 6 (15%) had CD. The frequency of iron deficiency anemia was also significantly higher in the CD group, and there was a significant association between IDA and CD (*P* = 0.01).

Multivariate logistic regression analysis showed that IDA, anti-TTG level, and *Helicobacter pylori* infection were independent predictors of positive histopathological findings of Marsh I-III, which is compliant with CD (Table 2). However, the only independent predictor of definitive CD, based on both positive serology and pathology, was IDA (odds ratio = 17.65, 95%CI = 1.538‐202.523, and *P* = 0.02).

## 4. Discussion

Dyspepsia is a common symptom that may be accompanied by a variety of digestive conditions [2, 11, 12]. Many patients with CD can present with dyspepsia [13], but the prevalence of CD in dyspeptic patients is reportedly around 0.5-2% [14–18]. The necessity of multiple duodenal biopsies for detection of CD in dyspeptic patients remains under controversy, and there is a paucity of evidence in this regard. Current guidelines suggest that routine biopsies of normal-appearing duodenal mucosa for diagnosis of CD should be justified and are indicated in patients with specific conditions [1]. We aimed to investigate the prevalence of CD in dyspeptic patients based on both serology and histology and find the factors associated with CD in these patients. We secondarily aimed to assess the necessity and efficacy of routine biopsies from normal-appearing duodenal mucosa in detecting CD in dyspeptic patients.

Prevalence of CD in patients with a family history of CD in first-degree relatives was 13.3%, which is higher than that of the general population (1%) [6, 7]. Moreover, recent ACG guidelines suggest that the incidence of CD is 20% in siblings and 10% in other first-degree relatives [1], which is in line with the results of this study. Our study showed bloating as the most common type of dyspepsia in CD patients. Other studies mention abdominal pain as the most common symptom in CD patients [12].

Abnormally high levels of anti-TTG were recorded in 6.8% of our dyspeptic patients. This finding is near the results of a previous study on western Iranian population, which reported 7% serological positivity for CD among patients with functional dyspepsia [24]. In a previous systematic review and meta-analysis of 15 studies, the prevalence of positive serology of CD among dyspeptic patients was reported to be 7.9% [25], which is comparable to our findings. However, both of the mentioned studies considered either endomysial antibody or anti-TTG as the diagnostic criteria for positive serology, while we only evaluated anti-TTG.

Our results showed the prevalence of positive histopathology for CD among dyspeptic patients to be 4.5%. The pooled prevalence of biopsy-proven CD was reported 1% (ranged between 0.8 and 2%) in studies that performed duodenal biopsy as the first-line method, as reported by Ford et al. in their meta-analysis [25]. This inconsistency might be due to the difference in ethnicity and characteristics of the patients.

We found the prevalence of definitive CD diagnosis to be 2.8% among our dyspeptic patients, which is higher than the reported figure for general population [6, 7]. Our findings are also slightly higher than the reported prevalence for CD among dyspeptic patients [14–18]. Lasa et al. compared 320 dyspeptic patients with 320 healthy individuals in Argentina and found the prevalence of CD to be 1.25% in patients with dyspepsia and 0.62% in healthy controls [26]. The inconsistencies between our results and those mentioned can be attributed to different patient characteristics as well as different diagnostic criteria for detecting dyspepsia and CD.

A number of factors may influence the decision to perform small bowel biopsy such as type of dyspepsia (e.g., bloating or abdominal pain), IDA, and a family history of CD. Using anti-TTG serology, 6.8% of the patients were diagnosed with CD, while 4.5% of patients were identified as CD using histopathological findings of duodenal samples (grades I-III of modified Marsh criteria). Finally, 2.8% of patients, who had both the serological and histopathological criteria, were diagnosed as definitive CD. Therefore, it can be concluded that performing biopsy of normal-appearing duodenal mucosa in dyspeptic patients during upper gastrointestinal endoscopy yields more specificity in comparison with serological tests.

This study shows that it is helpful to investigate CD through duodenal biopsy only in dyspeptic patients with the endoscopic markers of CD. A few studies recommend routine duodenal biopsy in normal-appearing mucosa while others did not [2, 16, 18] [27]. Nevertheless, Lecleire et al. state that endoscopic markers of villous atrophy are not able to identify a subgroup of patients who would benefit from duodenal biopsy [28]. Contrary to previous statements, Lasa et al. stated that screening in dyspeptic patients cannot be recommended due to comparable prevalence of CD in healthy individuals and dyspeptic patients [26].

Our research showed IDA, anti-TTG level, and *H*. *pylori* infection to be predictive factors for histological findings of Marsh I-III, while IDA was the only predictive factor for definitive diagnosis of CD in dyspeptic patients. *H*. *pylori* infection can lead to duodenal intraepithelial lymphocytosis, being reported as a major cause of lymphocytosis duodenosis, after CD [29]. Yet, studies have failed to discover any significant relationships between *H*. *pylori* and CD risk [30].

Anemia is the most common nongastrointestinal symptom of CD, and screening of CD has led to an increase in identification of the etiology behind IDA [1, 31]. In our sample of dyspeptic patients, 15% of anemic patients had CD. In contrast, the prevalence of biopsy-proven CD in IDA cases has been reported to be 3.2% in a recent systematic review [32]. The prevalence of CD has reportedly increased among the individuals with unexplained IDA, regardless of the presence of gastrointestinal symptoms [33]. It has been also reported that the severity of CD was higher in patients with anemia, compared with those without anemia. Therefore, timely diagnosis of CD is of high clinical importance.

Rather than limiting our sample size and performing HLA DQ2/DQ8 analysis, we included a large number of patients suffering from dyspepsia and tested for both serological and histological findings of CD. In cases with high anti-TTG levels and normal biopsy, endoscopy was repeated to increase the accuracy of findings. Moreover, in cases with abnormal pathology and normal serology, anti-TTG IgA levels were rechecked. Nevertheless, our study had some limitations. For instance, including healthy control subjects could have led to a better comparison. In addition, we did not perform endomysial antibody test, which could have added to the accuracy of serological diagnosis. Moreover, we could not follow the patients with high anti-TTG levels but normal biopsy findings, due to the scope of our objectives and limited time span of our research design. A prospective study that can follow such patients would add more insight.

## 5. Conclusion

To conclude, the results showed a higher prevalence of CD in our sample of dyspeptic patients compared to both general population and dyspeptic patients from other ethnic origins. IDA could be recommended as a predictor for CD. Particularly, CD should be considered in patients with abdominal pain, bloating-type dyspepsia, IDA, and those with a positive family history of CD; therefore, serological screening is recommended in these groups. However, since all patients with CD showed endoscopic markers of CD in duodenoscopy, it seems that routine duodenal biopsy from normal-appearing mucosa is not recommendable. Nonetheless, biopsy of normal-appearing duodenal mucosa may be feasible in patients with abnormal anti-TTG level, IDA, and *H*. *pylori* infection.

A key finding of this study is the importance of a preoperative serologic (anti-TTG) test, evaluation of serum ferritin, obtaining clinical and family history, and careful endoscopic evaluation. These steps can provide a cost-effective clinical guideline that will help improve the diagnostic yield of celiac disease in dyspeptic patients.

## Figures and Tables

**Table 1:** Comparison of the characteristics of participants with and without celiac disease.

Variable		Non-CD (*N* = 515)	CD (*N* = 15)	*P*
Demographics				
Age (years)		46.11 ± 15.2	43.27 ± 12.6	0.43^∗^
Sex	Male	159 (30.0%)	4 (26.0%)	0.48^∗∗^
	Female	356 (67.0%)	11 (73.0%)	
Medical history				
Dyspeptic complaint	Bloating	113 (23.3%)	7 (46.7%)	0.09^∗^
	Epigastric pain	205 (42.3%)	6 (40.0%)	
	Postprandial fullness	37 (7.6%)	2 (13.3%)	
	Early satiety	34 (7.0%)	0 (0.0%)	
	Reflux	96 (19.8%)	0 (0.0%)	
Family history of CD		1 (0.2%)	2 (13.3%)	<0.01^∗^
Laboratory findings				
Anti-TTG (U/ml)		3.22 ± 13.98	150.66 ± 116.97	<0.01^∗∗^
Abnormal anti-TTG level		21 (4.1%)	15 (100.0%)	<0.01^∗^
Hemoglobin (mg/dl)		13.32 ± 1.56	12.08 ± 1.31	<0.01^∗∗^
Ferritin (ng/ml)		95.20 ± 61.38	28.83 ± 20.29	<0.01^∗∗^
TIBC (*μ*g/dl)		315.77 ± 63.44	408.93 ± 48.25	<0.01^∗∗^
Iron deficiency anemia		34 (6.6%)	6 (40.0%)	<0.01^∗^
Vitamin D (ng/ml)		18.33 ± 11.78	17.14 ± 10.54	0.69^∗∗^
Vitamin D deficiency		329 (63.9%)	11 (73.3%)	0.32^∗^
Endoscopic findings				
*H. pylori* infection		2 (13.3%)	13 (86.7%)	0.57^∗^
Histopathological findings of the mucosal specimens	No atrophy	487 (98.4%)	0 (0.0%)	<0.01^∗^
	Marsh I	5 (1.0%)	3 (20.0%)	
	Marsh II	3 (0.6%)	3 (20.0%)	
	Marsh III	0 (0.0%)	9 (60.0%)	
Duodenoscopy findings	Normal	362 (73.3%)	0 (0.0%)	<0.01^∗^
	Atrophy	18 (3.6%)	7 (46.7%)	
	Scalloping	4 (0.8%)	1 (6.7%)	
	Nodularity	6 (1.2%)	3 (20.0%)	
	Fissuring	2 (0.4%)	3 (20.0%)	
	Ulcer	43 (8.7%)	0 (0.0%)	
	Erosion	59 (11.9%)	1 (6.7%)	

CD: celiac disease; TTG: tissue transglutaminase; TIBC: total iron binding capacity; H: Helicobacter. ^∗^Chi-square test. ^∗∗^Independent samples *t*-test.

**Table 2:** Multivariate logistic regression model for the prediction of celiac disease.

Predictor	Coefficient (*B*)	Odds ratio	95% confidence interval		*P*
			Lower bound	Upper bound	
Based on positive pathology (Marsh I-III)					
IDA	0.763	2.14	1.062	4.334	0.03
Anti-TTG	1.270	3.56	1.707	7.432	<0.01
*H. pylori* infection	1.425	4.16	2.224	7.780	<0.01
Based on both positive serology and pathology					
IDA	2.871	17.65	1.538	202.523	0.02

IDA: iron deficiency anemia; TTG: tissue transglutaminase; H: Helicobacter.

## Data Availability

The data regarding of this work is available upon reasonable request from the authors. Requests for data will be considered by the corresponding author, who can be contacted at GoshayeshiL@mums.ac.ir.
